# Automated Interpretation and Extraction of Topographic Information from Time of Flight Secondary Ion Mass Spectrometry Data

**DOI:** 10.1038/s41598-017-17049-y

**Published:** 2017-12-06

**Authors:** Anton V. Ievlev, Alexei Belianinov, Stephen Jesse, David P. Allison, Mitchel J. Doktycz, Scott T. Retterer, Sergei V. Kalinin, Olga S. Ovchinnikova

**Affiliations:** 1 The Center for Nanophase Materials Sciences, Oak Ridge National Laboratory, 1 Bethel Valley Rd., Oak Ridge, TN 37831 USA; 2 Institute for Functional Imaging of Materials, Oak Ridge National Laboratory, 1 Bethel Valley Rd., Oak Ridge, TN 37831 USA; 3 Biosciences Division, Oak Ridge National Laboratory, 1 Bethel Valley Rd., Oak Ridge, TN 37831 USA; 4 Department Biochemistry & Cellular & Molecular Biology, University of Tennessee, Knoxville, Tennessee USA

## Abstract

Time of flight secondary ion mass spectrometry (ToF-SIMS) is a powerful surface-sensitive characterization tool allowing the imaging of chemical properties over a wide range of organic and inorganic material systems. This technique allows precise studies of chemical composition with sub-100-nm lateral and nanometer depth spatial resolution. However, comprehensive interpretation of ToF-SIMS results is challenging because of the very large data volume and high dimensionality. Furthermore, investigation of samples with pronounced topographical features is complicated by systematic and measureable shifts in the mass spectrum. In this work we developed an approach for the interpretation of the ToF-SIMS data, based on the advanced data analytics. Along with characterization of the chemical composition, our approach allows extraction of the sample surface morphology from a time of flight registration technique. This approach allows one to perform correlated investigations of surface morphology, biological function, and chemical composition of *Arabidopsis* roots.

## Introduction

Time of Flight Secondary Ion Mass Spectrometry (ToF-SIMS) allows direct investigation of local chemical composition in a wide range of organic and inorganic material systems^1–10^. Within complex biological systems, ToF-SIMS, co-registered with techniques like atomic force microscopy, enables the observation of chemical changes in concert with changes in morphology and physical properties. In ToF-SIMS, the sample surface is bombarded by a primary focused ion beam, (*e.g*. Bi^+^) which causes a collision cascade in the top surface layer, yielding^11,12^ charged particles and clusters, which is then accelerated by a uniform electric field into a time-of-flight detector. At the detection stage, each element is represented by a peak with corresponding mass-to-charge ratio *m*/*z*, comprising a mass spectrum^13^. These focused ion beam measurements enable chemical composition studies in 2D and 3D, with sub-100 nm lateral resolution^14,15^, and monolayer sensitivity with the aid of an additional sputtering source.

ToF-SIMS data interpretation can be challenging, as acquired datasets are multidimensional, with multiple mass spectra measured along 2 or 3-dimensional grids. The total number of spectra can easily exceed 1 million for modern ToF-SIMS instruments with each spectrum consisting of multiple peaks, corresponding to a certain element or a molecular cluster. Additionally, these multidimensional datasets are often collected from samples having pronounced topographical features, which can introduce significant peak shifts that further confound automated data analysis^16–18^. Standard data analysis techniques typically involve simply finding the areas under manually selected peaks (based on prior knowledge of sample composition, or derived from an averaged spectrum), with the area of these peaks serving as a primary quantity for spatial signal mapping. However, these types of approaches have drawbacks, and there are better statistical methodologies where large quantities of data can be considered an asset. Using classical methods can result in crucial information loss if certain peaks have not been selected or detected. Secondly, averaging, or individual peak processing, precludes recovery of elemental spatial correlation. Therefore, if one does not treat the data set as a whole, then element coexistence needs to be checked manually through tedious comparison of multiple elemental maps and their spatial distribution.

An example of an alternative approach to data analysis can be realized by utilizing multivariate analysis (MVA) tools, consisting of supervised, or unsupervised machine learning. To date, these tools have been successfully applied for investigation and interpretation of scanning probe microscopy^19–21^, confocal-Raman spectroscopy^22–24^, ToF-SIMS^25–31^ and other data^32–34^. To take advantage of MVA, experimental data is converted into a set of mathematical vectors in a multidimensional space. In ToF-SIMS, a vector can be represented by a mass spectrum collected at a spatial point, or a voxel. However, the sheer number of spectral points in the raw mass spectrum, makes performing MVA of ToF-SIMS computationally intensive. To overcome this issue, coarse data pre-processing by binning^28^ (with bin width up to 1 *u*) or analysis of the preselected peaks of interest^27^ can ameliorate the processing load. However, these types of approaches suffer from a possible loss of spectral information, where the data pretreatment can significantly alter the final results.

In this work, we develop an approach for comprehensive interpretation of multidimensional ToF-SIMS data by utilizing multivariate statistical analysis and applying this approach to a challenging problem in biological imaging. Here, an *Arabidopsis thaliana* seedling root, which is non-planar and chemically complex, is analyzed by ToF-SIMS. We prepare the data using an automated data compression approach, retaining pertinent information without loss in spectral resolution. These data are processed further using principal component analysis (PCA), to identify regions of fundamentally different chemical compositions. Furthermore, our early results indicated a significant deleterious influence of the sample surface topography on the final mass spectra, which led to significant shift of the mass peaks and thus hampered automated data analysis. Therefore, we also present a theoretical framework to circumvent the error associated with secondary ion trajectories originating at different sample heights. This universal approach allows robust shift correction, and enables qualitative topography reconstruction solely from the ToF-SIMS data.

## Experiment and results

**Figure 1:**
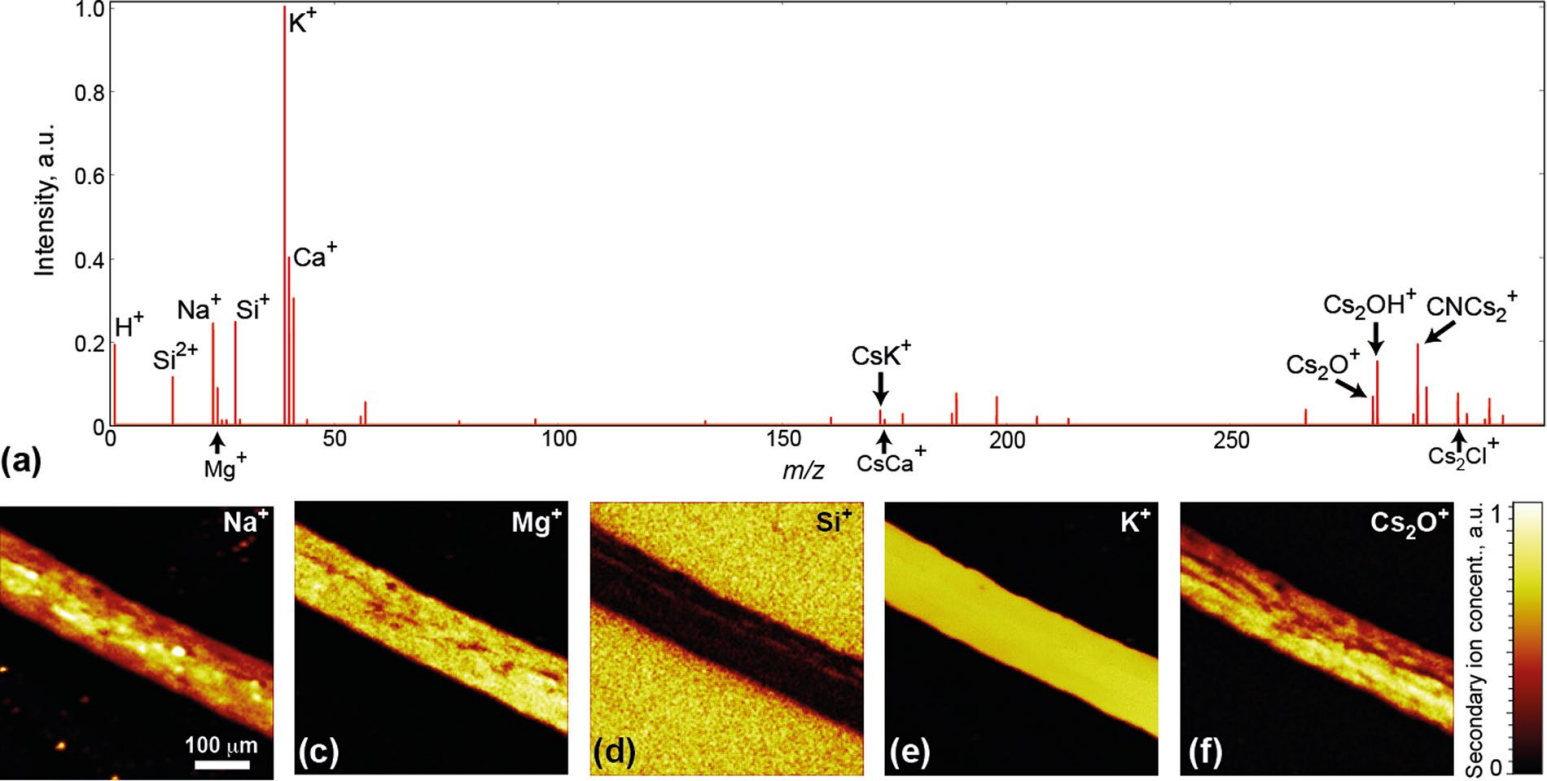
Classical approach to analysis of the ToF-SIMS data of the *Arabidopsis* root. (**a**) Averaged mass spectrum of *Arabidopsis* root. (**b**–**f**) Maps of the spatial distribution of elements, as specified in the figure.

We have chosen an *Arabidopsis* root immobilized and dried on a SiO_2_ substrate as our sample. The complex chemical composition of a growing root is expected to vary along the length of the root, reflecting different stages of root development, as well as across the root, reflecting differences between the inner and outer portions of the root. Moreover, the sample curvature, associated with the cylindrical architecture of the root, presents an interesting model system to address the challenges associated with assembling and analyzing data from samples with significant topographical complexity. ToF-SIMS measurements were performed using ToF.SIMS5 (IONTOF, Germany) mass spectrometer with a focused bismuth liquid metal ion gun as the ionization source, and cesium sputtering ion gun for sample cleaning and depth profiling. Vacuum level inside the chamber was around 5 × 10^−9^ mbar during the measurements. An averaged mass spectrum is shown in Fig. 1a. Chemical species common to biological systems, such as Na^+^, Mg^+^, K^+^_,_ and Ca^+^, as well as Si^+^ from the substrate and some small molecules with masses around 150–300 *u* are evident in these data. Although the measurements were performed in the positive ion detection mode, we were able to track some negative ions using peaks of the clusters formed with the sputtering Cs^+^ ions (e.g. Cs_2_O^+^, Cs_2_OH^+^, CNCs_2_^+^, Cs_2_Cl^+^). These species are also shown in the averaged mass spectrum. We intentionally excluded pronounced peaks of Cs^+^ and Cs_2_^+^ ions, as these are injected artificially into the sample. To study the spatial distribution of chemical species, the normalized area of selected peaks are plotted as a function of the spatial coordinates (Fig. 1b–f). Obviously, this approach for data interpretation is significantly limited by the characterization of the separate elements/peaks, while comprehensive analysis of the whole spectrum (more than 30 peaks clearly seen by eye) is impossible without statistical tools.

**Figure 2:**
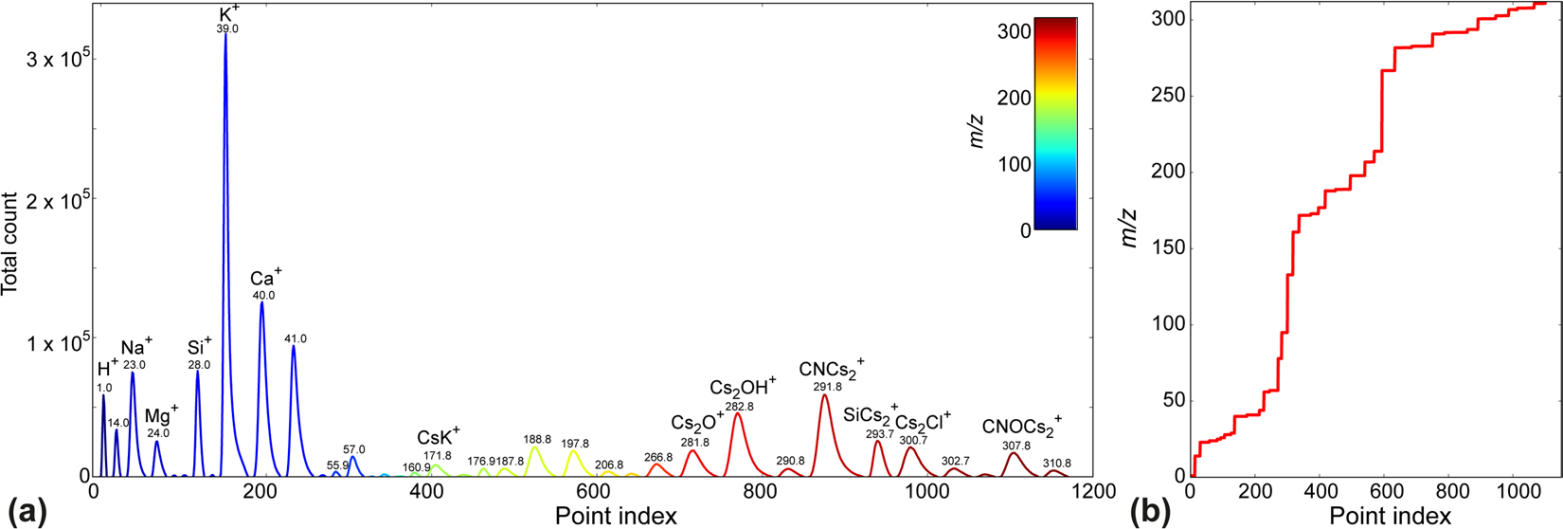
Data pre-processing for MVA analysis. (**a**) Shortened mass spectrum. (**b**) Calibration curve associating point index with actual mass.

Alternative MVA analysis of the ToF-SIMS data can be represented as a classical problem of dimensionality reduction, where vectors in the multidimensional space (individual ToF-SIMS mass spectra) are deconvoluted as a linear combination of a smaller number of eigenvectors, or endmembers. We use principal component analysis, (PCA) for the analysis of these data. However, processing the raw mass spec data is time consuming, as the length of the raw spectrum exceeds a million samples (100 μs of acquisition in the point with 50 ps time resolution) and as a consequence, the computational time is large. To resolve this issue, we developed an automated approach of spectrum compression without loss in spectral resolution. We start with an averaged mass spectrum (Fig. 1a) as a reference to identify mass segments corresponding to a useful signal defined as the total number of counts exceeding a preset threshold. This effectively reduces the overall number of data points, capturing only the useful, interpretable data. The original data is never altered, so the processes can be repeated with different count threshold values. Figure 2a illustrates the post-processed mass spectrum, plotted against a physically meaningless point index. The reader may note that the spectrum now contains 1000–2000 data points, making computation time reasonable. Figure 2b serves as a mass calibration map, assigning the point index to an actual *m/z* value. We used a count threshold level of 500 to 1000 counts, which roughly corresponds to a single ion detected in 65 spatial points, which is relatively speaking a very low value. Though this data representation may be considered more difficult for understanding and visual representation, it significantly increases the speed of MVA processing, without significant information loss.

**Figure 3:**
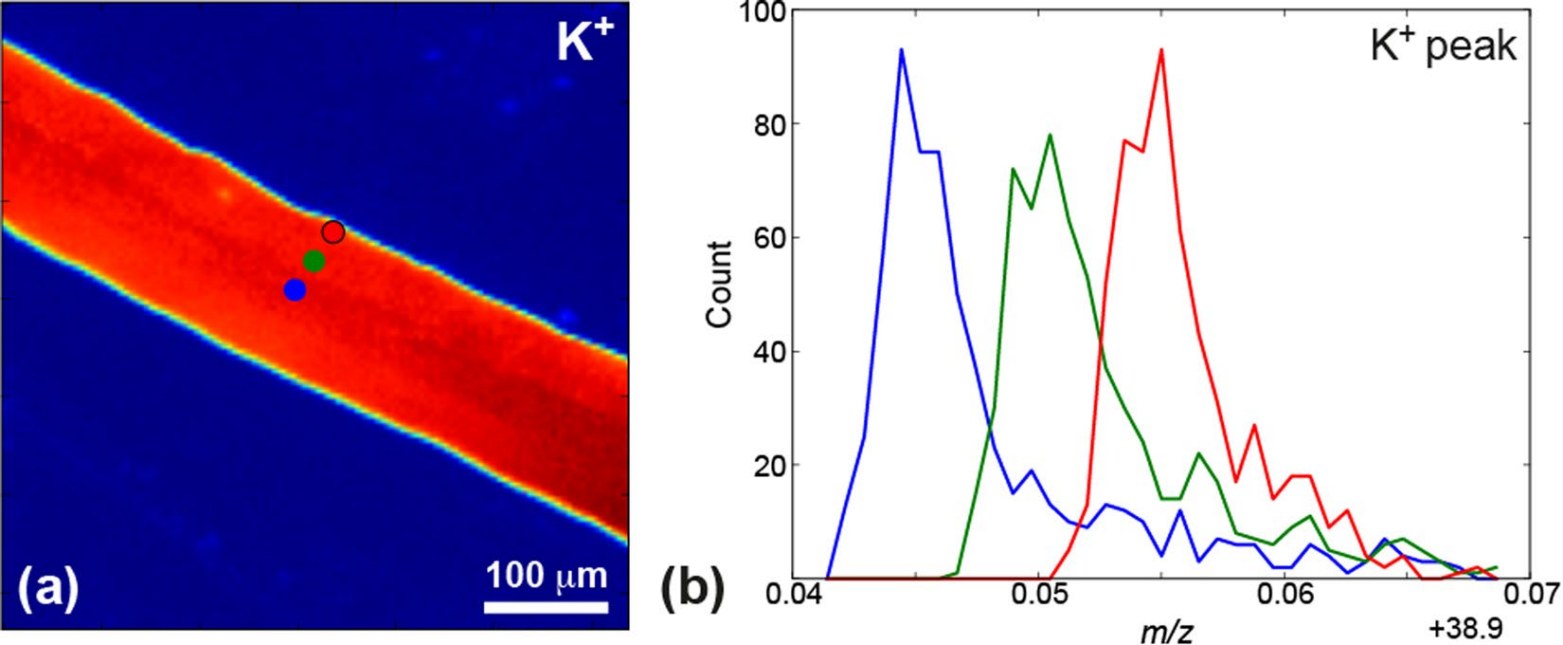
Topography induced shift of the mass spectra in time of flight mass spectrometry. (**a**) 2D map of K + distribution; (**b**) K^+^ peaks measured without shift correction in different positions, specified on the map (**a**).

To analyze compressed ToF-SIMS data, we further utilize PCA, which allows deconvolution of the mass spectrum into a linear combination of orthogonal eigenvectors, sorted by variance. In PCA the first few eigenvectors can be used to characterize the most salient features, while others mostly contain noise. The authors would like to note, that to make this a robust decision, a Scree plot is analyzed to determine the relative variance of each of the eigenvectors, as well as the overall variance distribution. Loadings, or abundance maps show the contribution of each of the principal components spatially (see *Supplemental Materials* for detailed information about PCA). Detailed analysis of PCA results performed over the preprocessed SIMS data (Supplemental Materials Fig. S1b) highlighted features mostly related to the sample morphology. Abundance maps of principal components 2–3 clearly show edges and the central part of the root. Simultaneously, close analysis of the corresponding eigenvectors shows characteristic peak shift behavior (positive to negative change for each peak). To confirm this notion, we plot the K^+^ peaks at three different points on the root surface, as illustrated by the blue, green, and red markers in Fig. 3a. As the reader may see, the data shows a difference of about 0.009 *u* (~3 ns in time of flight) for the K^+^ data measured at the central part of the sample as opposed to the sample edge (Fig. 3b).

This peak shift is related to the surface morphology of root, since the height at the edge and the center differ, thus yielding a different time of flight for the same chemical species. The experiment shows a decrease in the time of flight (mass-to-charge ratio) for ions collected from the elevated parts of the root, which is roughly 200 µm taller than the edge. This parasitic effect is well known in the ToF-SIMS community, and it leads to undesirable widening of the mass spectrum peaks^16–18^. Recent studies suggested a number of corrections^17^. In this work, we considered the origin of this shift and demonstrate an approach to correct for this height effect and also extract sample morphology in the process.

**Figure 4:**
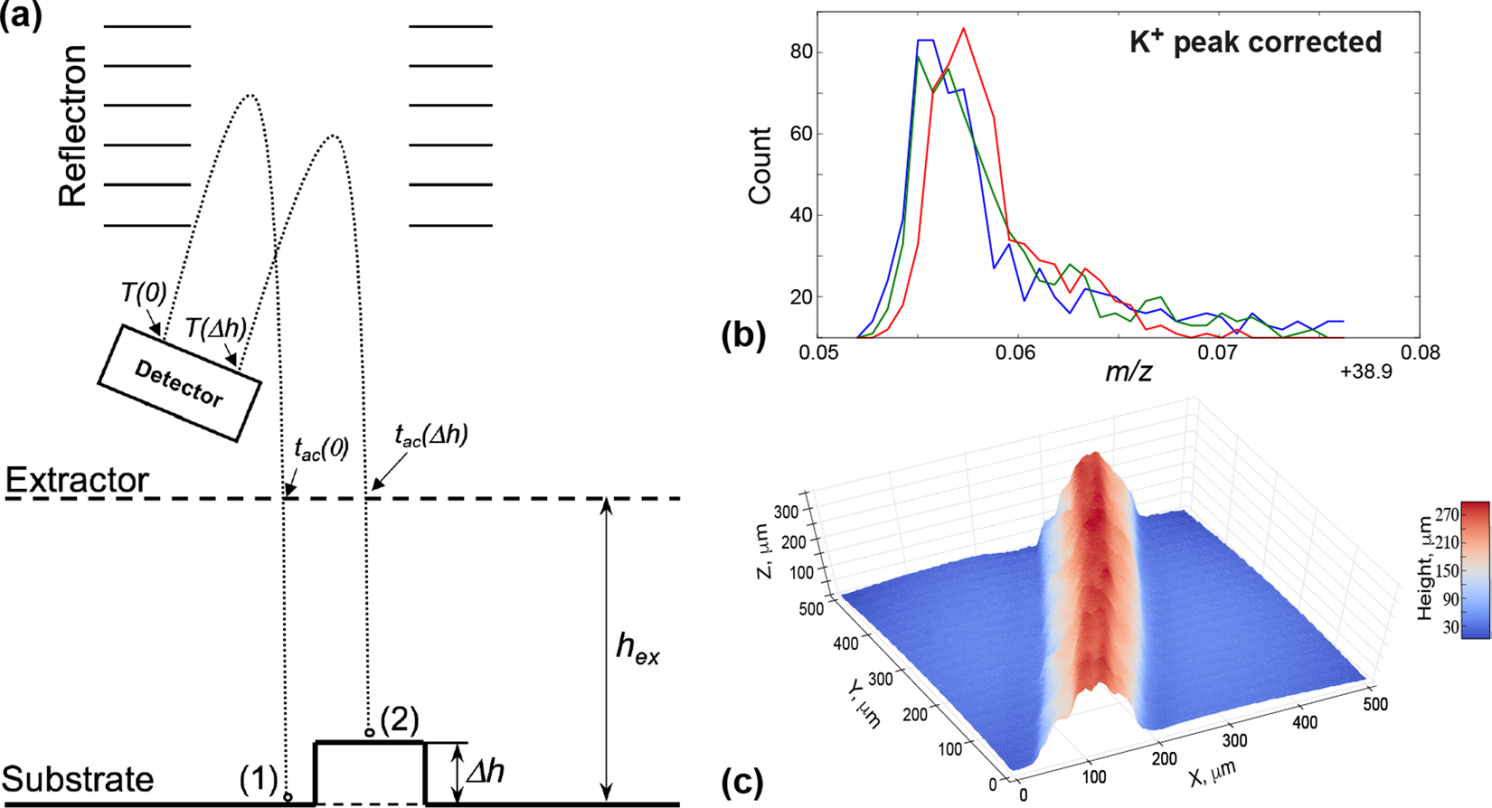
(**a**) Schematic of the ion motion in ToF-SIMS, (**b**) K^+^ peaks at points specified in Fig. 2 after shift correction and (**c**) Topography of the studied Arabidopsis root sample, calculated from shift of the Cs peak.

We considered a simplified version of the ToF-SIMS analyzer (Fig. 4a), where ions released from the sample surface are accelerated by the uniform electric field in the gap between the sample and the biased extractor (*U*_*ex*_ = 2 kV). Sample to extractor distance, in our experiments is *h*_*ex*_ = 1.5 mm. In this case, time of the K^+^ ion acceleration in a homogeneous electric field can be estimated as: ${t}_{ac}=\sqrt{\frac{2{h}_{ex}^{2}{m}_{K}}{{U}_{ex}e}}\approx 30.2\,ns$, where *m*_*K*_ is K^+^ ion mass and *e* – electron charge, while the total effective traveled path of the ion released from the surface is roughly *H*_*ef*_ = 2 m and total time of flight measured for K^+^ ions is *T*_*K*_ = 20.545 μs.

From Fig. 4a we can compare two ion paths with the same mass *m*: (*p*_1_) released from SiO_2_ substrate and (*p*_2_) released from the root at height *Δh* above the substrate. We provide a detailed description of the model as well as full mathematical calculations in the *Supplemental Materials*. Here we will discuss only the most significant conclusions.

At the extractor, the velocity of the ion released from the substrate, ${v}_{ex}(p1)=\sqrt{\frac{2{U}_{ex}e}{m}}$, will be higher than the velocity of the ion released from the root, ${v}_{ex}({\rm{\Delta }}h)={v}_{ex}(p2)\sqrt{\frac{{h}_{ex}-{\rm{\Delta }}h}{{h}_{ex}}}$. This difference will be, however, compensated by the reflectron. On the other hand, the acceleration time spent by the second ions to travel the distance from the root to extractor will also be smaller: ${t}_{ac}({p}_{2})={t}_{ac}({p}_{1})\sqrt{\frac{{h}_{ex}-{\rm{\Delta }}h}{{h}_{ex}}}$. This difference cannot be easily compensated and will result in a topographically induced shift, observed experimentally (Fig. 3b). The total time of flight of the second ion *T(p*_2_) can be calculated using the following expression, given by Equation 1:

1 $$T({p}_{2})=T({p}_{1})-{t}_{ac}({p}_{1})(1-\sqrt{\frac{{h}_{ex}-{\rm{\Delta }}h}{{h}_{ex}}})$$

where *T(p*_1_) is total time of flight of the first ion, released from the substrate.

To establish the technique for the shift correction, we also have to consider the ions of different masses *m*_*x*_, starting from height *Δh*, with the total time of flight *T*_*x*_(*Δh)* presented in terms of *T(0)* and *T(Δh)* by Equation 2:

2 $${T}_{x}({p}_{2})={T}_{x}({p}_{1})\frac{T({p}_{2})}{T({p}_{1})}$$

where *T*_*x*_ *(0)* is the time of flight of ion with mass *m*_*x*_ released from the substrate.

With equation (2) one can establish a universal correction procedure. One of the peaks in the mass spectrum serves as a reference, which allows the calculation of the correction factor *D* and its spatial distribution, given by Equation 3:

3 $$D(x,y)=\frac{{T}_{ref}^{sub}}{{T}_{ref}(x,y)}$$

here *T*_*ref*_^*sub*^ is the time of flight of the reference ions, and *T*_*ref*_(*x*, *y*) is the time of flight of the reference ion at a spatial point with coordinates (*x*, *y*).

The maximal measured time of flight in the dataset can be used as an estimate for *T*_*ref*_^*sub*^. In this case, times of flight of all ions (besides the reference) can be corrected by Equation 4:

4 $$T^{\prime} (x,y)=T(x,y)\times D(x,y)$$

where T and T′ are uncorrected and corrected times of flight respectively.

Furthermore, the referenced peak position can also be used to quantitatively assess the surface topography, given by Equation 5:

5 $${\rm{\Delta }}h(x,y)={h}_{ex}(1-{(\frac{{t}_{ac}(0)-{\rm{\Delta }}T(x,y)}{{t}_{ac}(0)})}^{2})$$

where *ΔT*(*x*, *y*) = *T*_*ref*_^*sub*^ − *T*_*ref*_(*x*, *y*). The value of *t*_*ac*_(*0*) cannot be directly measured and needs to be estimated using known instrument parameters.

In our work, we used the Cs^+^ peak as a reference to calculate the correction factor *D* (Fig. 3c). This peak has been used as it has maximal intensity from collected mass spectra. However, any of the peaks can be used for correction, as demonstrated by theoretical calculations. The final deviation of the K^+^ ions after the topographic correction did not exceed 1.5×10^−4^ *u* (Fig. 4b). The estimated topography of the root with a line height profile is shown in Fig. 4c. This approach of topography extraction can be used as a universal technique for ToF-SIMS data interpretation. We expect it to be applicable for surface morphology features from about 10 μm to hundreds on microns. Detection of the smaller features would be problematic due to small values of spectral shift. For instance, K^+^ time of flight shift for studied sample was around 3 ns, with detection accuracy of 50 ps, which gives minimal detectable feature height of about 5 μm. Measurements of samples with much bigger features will introduce additional problems of non-uniform sputtering and inefficiency of the charge compensation.

Correction of the time of-flight shift allowed us to proceed with data analysis and decouple chemical and topographic channels convoluted in the raw ToF-SIMS data. Corrected data in this case, can be processed using MVA for chemical interpretation. To do this we performed PCA over the corrected dataset (Fig. 4), by utilizing the Bellerophon Environment for Analysis of Materials – BEAM^35^. BEAM is a computational workflow software, which can be run on a wide range of operating systems. This lightweight application enables access to High Performance Computing (HPC) platforms easy by offering an intuitive graphical-user-interface, a choice of scalable data analysis algorithms, simulation packages, input and output data storage, as well as data sharing capabilities.

In the analysis, the K^+^ peak, along with Cs^+^ and Cs_2_^+^ peaks have been excluded. The reasons for exclusion of the K^+^ peak was saturation, seen in the spatial distribution maps. Cs^+^ and Cs_2_^+^ peaks have also been excluded as they originate from the sputtering source and are not expected in the root chemical composition.

**Figure 5:**
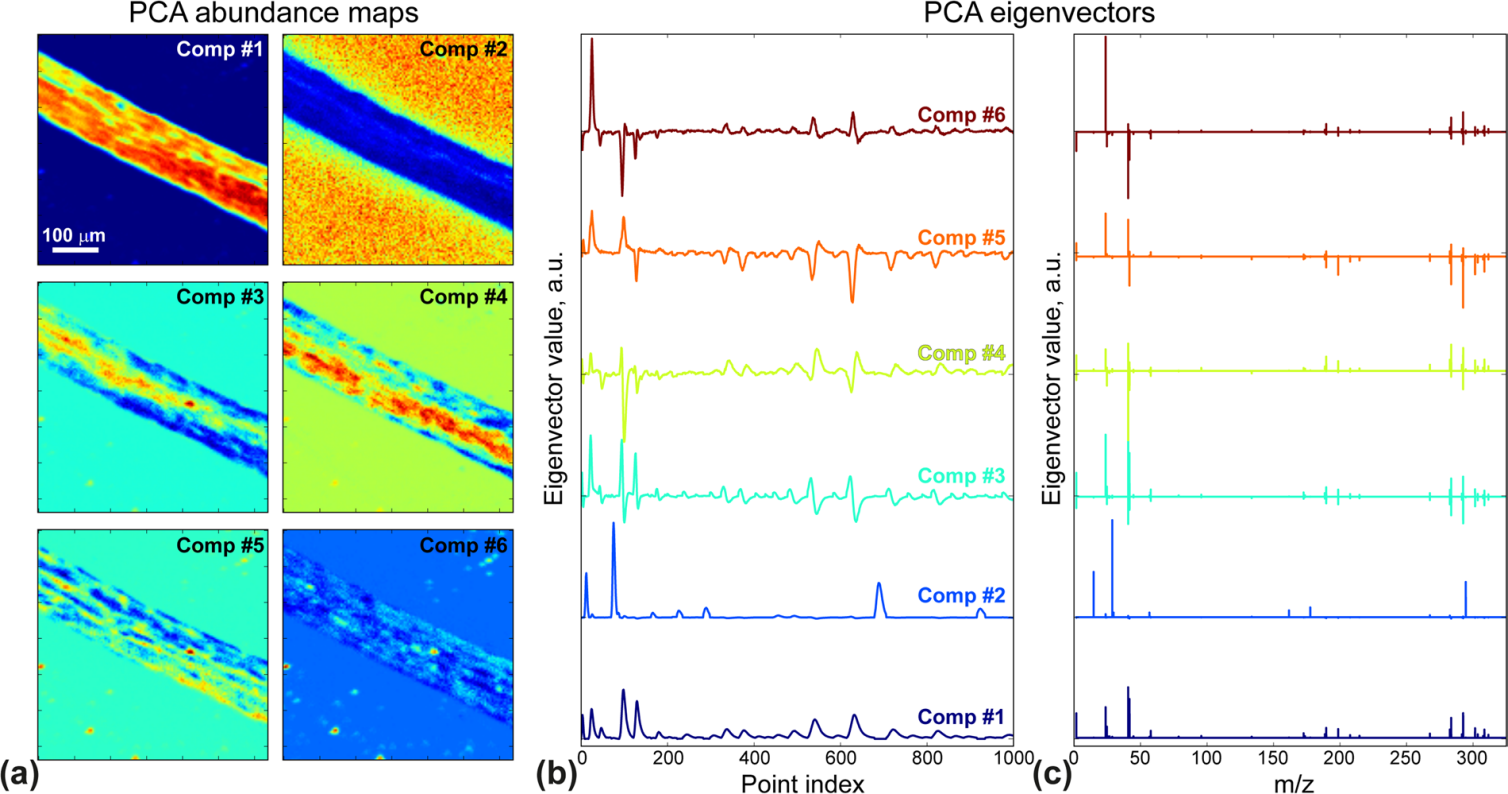
Principal component analysis of ToF-SIMS data. (**a**) Abundance maps and (**b**,**c**) eigenvectors plot *versus* (**b**) point index and (**c**) mass-to-charge ratio.

To interpret PCA results one should remember that the mass spectrum at each point represents a linear combination of eigenvectors with loading coefficients illustrating the abundance of each of the eigenvectors. Figure 5a are the loading coefficients for PCA eigenvectors 1–6, shown as a function of the point index in Fig. 5b, and the *m/z* in Fig. 5c. For non-whitened data, the first PCA component is the averaged mass spectrum of the root, without the characteristic Si peaks. The second component, on the other hand, is Si signal dominated, showing highest abundance for Si peaks, (Si^+^, Si^2+^, Si_2_^+^, *etc*.) found outside the root on the substrate (Fig. 5a Comp #2). Component #3 shows regions with higher concentration of K^+^ and Ca^+^, while component #5 shows Na^+^ inclusions. Component #4 demonstrates pronounced peaks with masses above 150 *u*, corresponded loading map shows regions of the higher concentration of small molecules and clusters. Finally, component #6 is most likely a signature of contamination, which is sparsely distributed over the root and substrate. The distribution of these eigenvectors allows differentiation of background from the sample, and the exciting possibility of distinguishing chemical characteristics that correlate with structural features of the root.

It needs to be noted that in the ToF-SIMS instrument, the ion beams are tilted with respect to the sample surface, which may cause inhomogeneity of sputter yields on samples with pronounced surface morphology. The magnitude of this phenomenon is defined by the sample/ions interaction. In the studied case of *Arabidopsis*, PCA maps as well as topography profile do not show any preferential areas associated with sputtering direction, which demonstrates that shading effect is small. However, in the general case one needs to be aware of possible artifacts caused by the tilt of the ion guns.

## Conclusion

In this work we developed an automated approach to comprehensively interpret multidimensional mass spectrometry data based on multivariate analysis. We applied this methodology to characterize a sample of *Arabidopsis* root, studied with ToF-SIMS. Our raw results show mass spectrum features (*i.e*. peak shift) related to the surface morphology of the sample, which suppresses the application of multivariate analysis, and yields erroneous results. Therefore, we modeled the travel behavior of the secondary ions inside the ToF-SIMS analyzer. This allowed us to establish a universal correction technique and estimate surface topography of the sample by only using the ToF SIMS data.

Data analysis performed using Principal Component Analysis identified regions along the root with different chemical composition. This demonstrates the opportunity to use ToF-SIMS to correlate chemical characteristics with functional processes on biological samples with complex morphologies. This approach can be used as a basis for universal topography correction in ToF-SIMS data, as well as comprehensive interpretation of the mass spectrometry data in 2- and 3-dimensions.

## Methods

### Studied samples

In our measurements, we used a biological sample of *Arabidopsis* root deposited on a SiO_2_ substrate. *Arabidopsis thaliana* seeds were purchased from Lehle seeds (PO Box 2366, Round Rock, TX, 78680–23666). Surface sterilization of seeds was accomplished by using either ethanol + Triton × 100 (1 Nelson 2009) or by a combination of ethanol and bleach+1% SDS (2 Choe 2001). The seeds were grown in sterile liquid growth media composed of 1.1 g of Murashige and Skoog basal Medium with “Vitamins” (3 Murashige and Skoog 1962), 0.5 g MES hydrate (4-Morpholineethanesulfonic acid hydrate) and 2.5 g of sucrose in 1 liter of distilled water. Approximately 20 to 40 sterilized seeds were placed in sterile culture dishes with growth media and placed near a window in the laboratory where the temperature could vary between 22–25 °C. Generally, the seeds would germinate in 2–3 days and would be used in experiments 4–6 days after germination. *Arabidopsis* root samples 0.5 to 1 cm in length were cut from plants growing in growth media and placed on the SiO_2_ samples and allowed to dry prior to analysis.

### Time of Flight Secondary Ion Mass Spectrometry

Mass spectrometry measurements were performed in positive ion detection mode using TOF SIMS-5 (ION-TOF GmbH, Germany) with a 30 keV Bi ion gun as the primary source with a focused ion beam spot size of ~5 μm, and a *m/Δm* resolution of 5000 ÷ 11,000. The imaging was performed across 500 × 500 μm areas of 256 × 256 points with a total acquisition time of 300 s. Cs^+^ ion-sputtering source operated at 1 keV and 50 nA across 700 × 700 μm area has been used for cleaning of the sample surface during the measurements. Low energy electron flood gun has been used during ToF-SIMS measurements to compensate accumulation of the positive charges on the sample surface.

## Electronic supplementary material

Supplementary Information
