# Supplementary material for: Automated Interpretation and Extraction of Topographic Information from Time of Flight Secondary Ion Mass Spectrometry Data
Source: Sci Rep. 2017 Dec 6;7:17099. doi: 10.1038/s41598-017-17049-y (PMC5719033; doi:10.1038/s41598-017-17049-y)
Supplement: Supplementary file 1 — Supplementary Information [file 41598_2017_17049_MOESM1_ESM.pdf]

Supplemental Materials

Automated Interpretation and Extraction of Topographic Information from Time of Flight Secondary Ion Mass Spectrometry Data

Anton V. Ievlev[1,2,*], Alexei Belianinov[1,2], Stephen Jesse[1,2], David Allison[4], Mitchel Doktycz[1,3], Scott Retterer[1,3], Sergei V. Kalinin[1,2] and Olga S. Ovchinnikova[1,2]

[1]The Center for Nanophase Materials Sciences, Oak Ridge National Laboratory, 1 Bethel Valley Rd., Oak Ridge, TN 37831

[2]Institute for Functional Imaging of Materials, Oak Ridge National Laboratory, 1 Bethel Valley Rd., Oak Ridge, TN 37831

[3]Bioscience Division, Oak Ridge National Laboratory,1 Bethel Valley Rd., Oak Ridge, TN 37831

[4]Dept. Biochemistry & Cellular & Molecular Biology, University of Tennessee, Knoxville, Tennessee, USA

*Corresponding Author:
Anton V. Ievlev
Center for Nanophase Materials Sciences
Oak Ridge National Laboratory, Oak Ridge, TN, 37831
ievlevav@ornl.gov

Supplemental Section 1. Detailed description of Principal Component Analysis

To allow PCA on the ToF SIMS 3- or 4- dimensional spectroscopic dataset consisting of *NxMxM* spatial (where N – number of points along z-direction; M – number of points along x and y directions) and S spectral points is transformed into the set of $N*M^2$ vectors A_i, each of length S.

Further, PCA deconvolutes each vector on a linear combination of the orthogonal, linearly uncorrelated eigenvectors v_k such that:

$$A_i = l_{ik} v_k , \tag{1}$$

Where l_{ik} are expansion coefficients, or component loadings, the eigenvectors v_k and the corresponding eigenvalues λ_k can be found from the singular value decomposition of the covariance matrix, $\mathbf{C} = \mathbf{A}\mathbf{A}^T$, where $\mathbf{A}$ is the matrix of all experimental data points $\mathbf{A}_{ij}$ i.e. the columns of $\mathbf{A}$ correspond to spectral vectors ($j = 1,..,M$), and rows correspond to spatial points, $i = 1,..,N^2$. The eigenvectors v_k (Fig. S1b) are orthogonal and are arranged such that the corresponding eigenvalues are placed in a descending order by variance. This means that first eigenvector v_1 contains the most important spectral information; the second v_2 contains the most important response after the subtraction of variance from the first one, and so on. In this manner the most relevant information about mass spectra can be characterized by a few first eigenvectors v_k while remaining eigenvectors are dominated by noise and random features of individual spectra.

PCA of big amounts of the data (up to 100GB) have been performed using capabilities of the supercomputer at Oak Ridge National Laboratory. This has been realized by utilizing the Bellerophon Environment for Analysis of Materials – BEAM. BEAM is a computational workflow managing software, available on Apple, Android, Linux, and Windows. This lightweight Java application is designed to make High Performance Computing (HPC) platforms easy to use by offering an intuitive graphical-user-interface, a choice of scalable data analysis algorithms,

simulation packages, input and output data storage, as well as data sharing capabilities; all with a click of a button (Fig. S2).

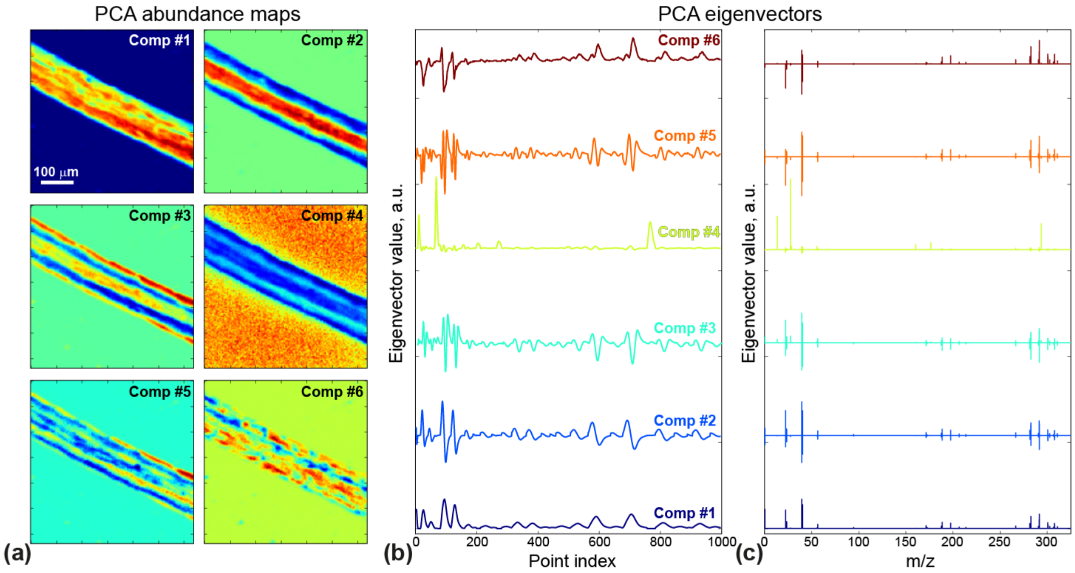

Figure S1. Principal component analysis performed on ToF SIMS data without correction of the topography induced peaks shift. (a) Abundance maps and (b-c) eigenvectors plot vs (b) point index and (c) mass-to-charge ratio.

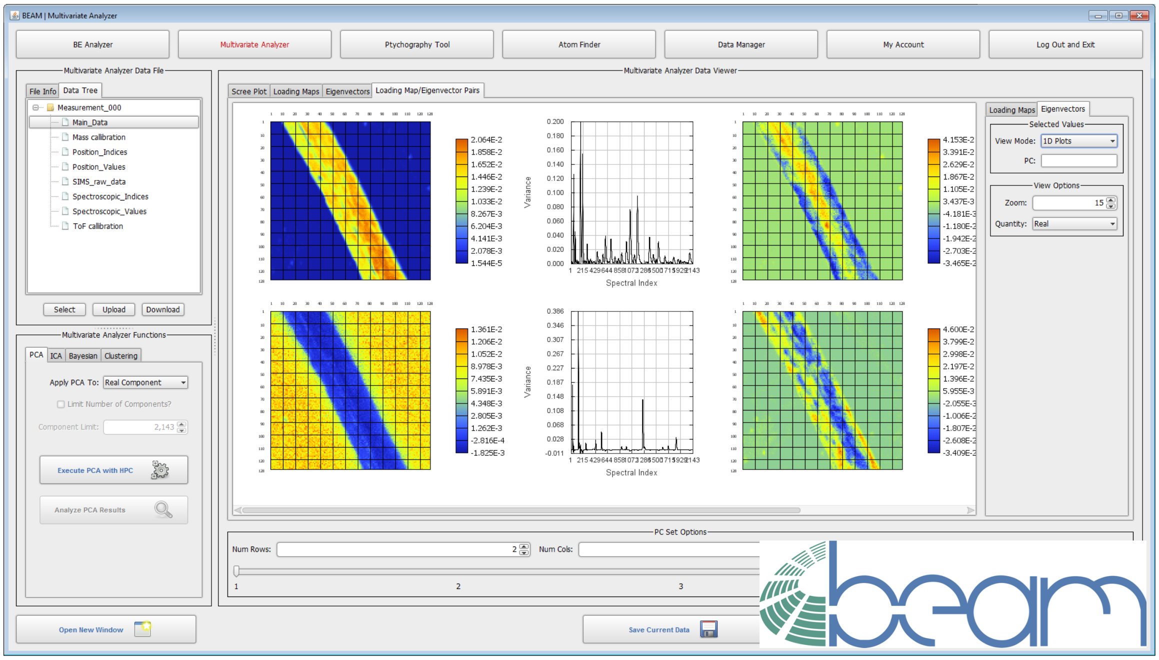

Figure S2. Interface of BEAM program, allowing universal and fast way of multivariate analysis, including PCA. of a wide range of microscopic and spectroscopic data

Supplemental Section 2. Calculation of the ions path in the ToF SIMS

To estimate difference in the time of flight induced by the topography of the studied sample and establish its correction technique, we consider simplified scheme of the ToF mass spectrometer (Fig. S3a). Ions released from the surface are accelerated in the uniform electric filed, produced in between biased extractor (U_{ex} = 2 kV) and grounded substrate. Distance between extractor and substrate h_{ex} = 1.5 mm is taken from the specification of the used ToF SIMS. After passing through the extractor ions drift to a reflectron, which is designed for equalization in the times of flight for ions of the same masses, but different initial kinetic energy (velocity). This is realized through the energy focusing in the time space. A total effective path of extracted ion in the used ToF SIMS is about H_{tot} = 2 m.

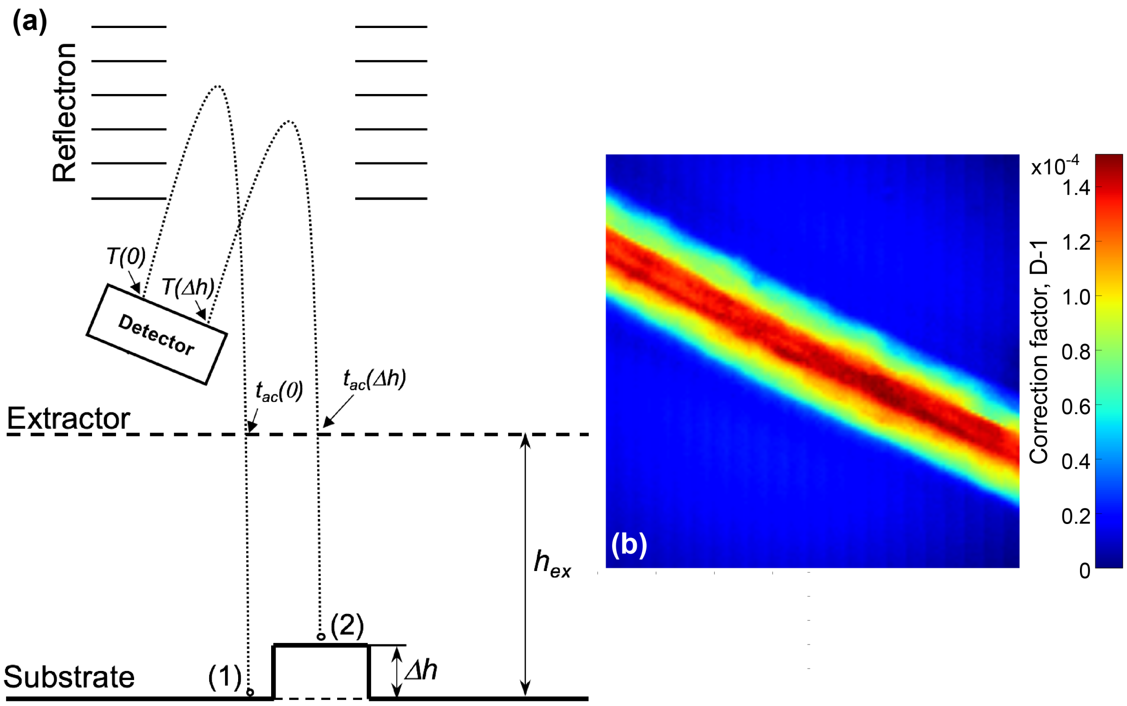

Figure S3. (a) Scheme of the ion motion in ToF SIMS. (b) Map of correction factor D, calculated from shift of the Cs peak.

In our calculations we first consider two ions of mass m: (1) is released from the substrate at the distance h_{ex} from extractor, (2) is released from the root surface Δh above the substrate with distance to the extractor equal $h_{ex} - \Delta h$. Velocities of the first and second ions at extractor are labeled as $v_{ex}(0)$ and $v_{ex}(\Delta h)$ respectively, their values can be found using following expressions:

$$v_{ex}(0) = \sqrt{\frac{2U_{ex}e}{m}} \tag{S1}$$

$$v_{ex}(\Delta h) = \sqrt{\frac{2U_{ex}e(h_{ex}-\Delta h)}{mh_{ex}}} = v_{ex}(0)\sqrt{\frac{h_{ex}-\Delta h}{h_{ex}}} \tag{S2}$$

where e is the electron charge and m – ion mass.

Acceleration times required for both ions to reach the extractor $t_{ac}(0)$ and $t_{ac}(\Delta h)$:

$$t_{ac}(0) = \sqrt{\frac{2h_{ex}^2 m}{U_{ex} e}} \quad \text{(S3)}$$

$$t_{ac}(\Delta h) = \sqrt{\frac{2h_{ex}(h_{ex}-\Delta h)m}{U_{ex} e}} = t_{ac}(0)\sqrt{\frac{h_{ex}-\Delta h}{h_{ex}}} \quad \text{(S4)}$$

Analyzer scheme with reflectron enabled compensation of the difference in the initial velocities, but variation in the acceleration time cannot be easily compensated and leads to the experimentally observed shift in the times of flight. In this case total time of flight *T* for the both ions can be estimated as:

$$T(0) = t_1^{ac} + t_r \quad \text{(S5)}$$

$$T(\Delta h) = t_2^{ac} + t_r \quad \text{(S6)}$$

where t_r time both ions spent to pass distance from the extractor to the detector.

Expression for the time of flight of the second ion can be rewritten, using (S3) and (S4):

$$T(\Delta h) = T(0) - (t_{ac}(0) - t_{ac}(\Delta h)) = T(0) - t_{ac}(0)\left(1 - \sqrt{\frac{h_{ex}-\Delta h}{h_{ex}}}\right) \quad \text{(S7)}$$

To establish correction procedure, we need to find how time-of-flight depends on the mass of the studied ion. To do that we consider another with a mass m_x, release from the root (height Δh). Its time of flight can be found by equation:

$$T_x(\Delta h) = T_x(0) - t_{ac}^x(0)\left(1 - \sqrt{\frac{h_{ex}-\Delta h}{h_{ex}}}\right) \quad \text{(S8)}$$

where T_x is corresponding ToF and $t_{ac}^x(0) = \sqrt{\frac{2h_{ex}^2 m_x}{U_{ex} e}}$.

Equation (S8) can be rewritten, using general form of (S3):

$$T_x(\Delta h) = T_x(0) - t_{ac}(0)\left(1 - \sqrt{\frac{h_{ex}-\Delta h}{h_{ex}}}\right)\sqrt{\frac{m_x}{m}} \quad \text{(S9)}$$

On the other hand, basic principles of the ToF SIMS provide proportionality pf the times of flight for ions of different masses: $\frac{T_x(0)}{T(0)} = \sqrt{\frac{m_x}{m}}$. Using this calibration and (S7):

$$T_x(\Delta h) = T_x(0) - \big(T(0) - T(\Delta h)\big)\frac{T_x(0)}{T(0)} = T_x(0)\frac{T(\Delta h)}{T(0)} \quad \text{(S10)}$$

Equation (S10) suggests approach for the correction of the time of flight shift, induced by the topography of the studied sample. To do that one can select one of the peaks in the mass spectrum as a reference and calculate spatial distribution of the correction factor:

$$D(x,y) = \frac{T_{ref}^{sub}}{T_{ref}(x,y)} \quad \text{(S11)}$$

where T_{ref}^{sub} is time of flight of the referenced ions from substrate and $T_{ref}(x,y)$ – time of flight of referenced ion in the point with coordinates *(x,y)*.

Maximal measured time of flight in the dataset can be used as an estimation for T_{ref}^{sub}. In this case times of flight of all ions (not only referenced one) can be easily corrected:

$$T'(x,y) = T(x,y) \times D(x,y) \quad \text{(S12)}$$

where T and T′ are uncorrected and corrected times of flight respectively.

Furthermore, times of flights for referenced ions can be used for estimation of the surface topography of the studied sample. Equation (S7) can be transformed by the following way:

$$\Delta h(x,y) = h_{ex}\left(1 - \left(\frac{t_{ac}(0) - \Delta T(x,y)}{t_{ac}(0)}\right)^2\right) \quad \text{(S13)}$$

where $\Delta T(x,y) = T_{ref}^{sub} - T_{ref}(x,y)$.

However, value of $t_{ac}(0)$ cannot be easily measured and needs to be estimated using parameters of spectrometer and eq. (S3). Extracted topography data of the studied Arabidopsis root is presented in figure S4.

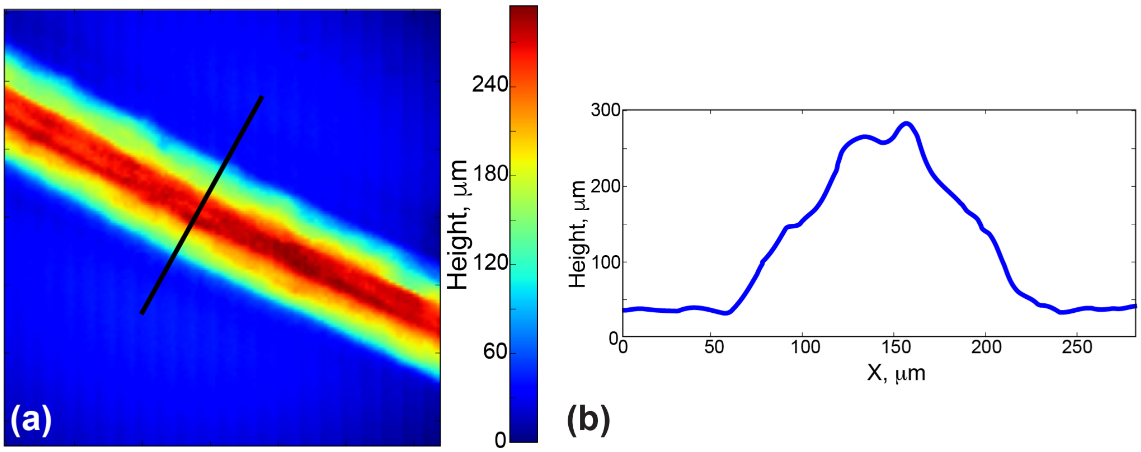

Figure S4. (a) Topography of the studied Arabidopsis root sample, calculated from shift of the Cs peak and (b) profile of root as labeled.
